# Rectal endometriosis causing colonic obstruction and concurrent endometriosis of the appendix: a case report

**DOI:** 10.1186/1752-1947-5-320

**Published:** 2011-07-20

**Authors:** N Katsikogiannis, AK Tsaroucha, K Dimakis, E Sivridis, CE Simopoulos

**Affiliations:** 1 Surgical Department, National System of Health, Hospital of Alexandroupolis, 68100 Alexandroupolis, Greece; 2 Second Department of Surgery, Medical School, Democritus University of Thrace, Dragana, 68100 Alexandroupolis, Greece; 3 Department of Pathology, Medical School, Democritus University of Thrace, Dragana, 68100 Alexandroupolis, Greece

## Abstract

**Introduction:**

Endometriosis is a clinical entity which presents with functioning endometrial tissue at sites outside the uterus. Bowel endometriosis is usually asymptomatic, but it may show non-specific symptoms. The presence and/or association of appendiceal endometriosis, concomitant with rectal endometriosis, is possible.

**Case presentation:**

A 36-year-old Greek woman was admitted to the emergency room of our hospital with signs of acute abdomen. On physical examination, our patient had a painful distended abdomen. Digital examination revealed an empty rectum and bowel obstruction was diagnosed. Our patient underwent exploratory laparotomy and rectum stenosis (almost complete obstruction) was observed. The bowel stenosis was resected, and temporary colostomy and appendectomy were performed. The pathology report showed endometriosis of the colon and the appendix, and our patient received medical treatment for endometriosis. Six months after this operation our patient had another surgery for restoration of large bowel continuity. No endometriosis was found. Our patient was doing well at the one-year follow up.

**Conclusion:**

Endometriosis of the bowel is a disease that may cause large bowel obstruction. In women of reproductive age, the surgeon should consider endometriosis as a differential diagnosis in case of various gastrointestinal symptoms.

## Introduction

Endometriosis is a clinical entity, which was first described by von Rokitansky Kitansky as the presence of functioning endometrial tissue at sites outside the uterus [1,2]. Endometriosis occurs in 3-10% of the general female population of reproductive age, 40-80% present symptoms such as pelvic pain, infertility, or both [2,3]. Endometriosis rarely involves the small intestine, the appendix, the colon, the lung or other tissues [4,5].

Bowel endometriosis is usually asymptomatic, but it may show non-specific symptoms, such as abdominal colic-like pain, nausea, vomiting, and general symptoms of intestinal obstruction [6,7]. Circumferential endometriosis of the rectum should be differentially diagnosed from inflammatory or malignant diseases [5]. Endometriosis of the appendix usually presents with abdominal pain [8]. The presence and/or association of appendiceal endometriosis, concomitant with rectal endometriosis, is possible, because endometriosis could occur in more than one anatomical location at the same time.

A PubMed search revealed less than 20 reported cases of large bowel obstruction due to endometriosis in the last 10 years. In none of these reports was the appendix involved. We add here an additional case report. We present a case of rectal endometriosis and bowel obstruction, together with appendiceal endometriosis, diagnosed after surgical treatment in a female patient of reproductive age.

## Case report

**Figure 1:**
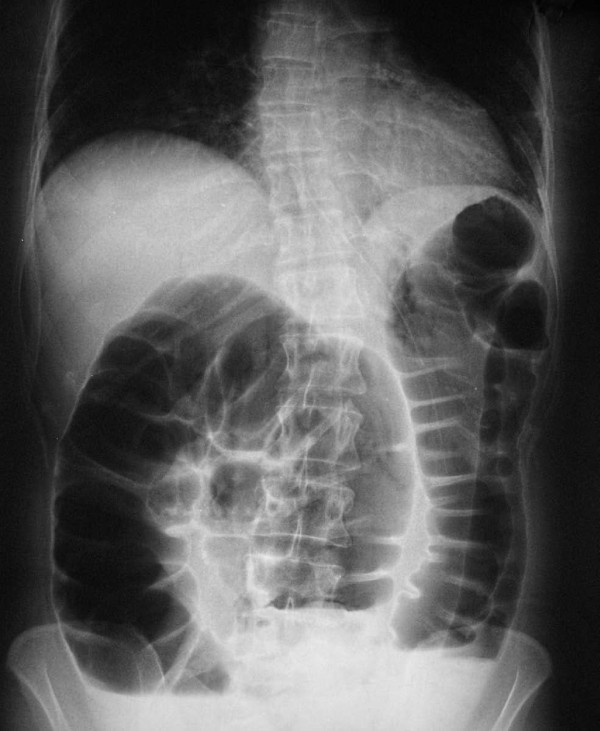
**Abdominal X-ray shows dilatation of proximal bowel segments**.

A 36-year-old Greek woman was admitted to the emergency room of our hospital with signs of acute abdomen. At admission, the physical examination revealed abdominal pain and abdominal distention. Our patient also complained of constipation and failure to pass gas or feces for three days. On physical examination, our patient had a painful distended abdomen. Digital examination revealed an empty rectum. An X-ray of her abdomen revealed a bowel obstruction (Figure 1). The impression was that the large bowel obstruction was due to a tumor.

Our patient had undergone a cesarean section for delivery of her first child, 12 years before this admission, in another country. One year after the cesarean, she was operated on for symphysiolysis because of an acute bowel obstruction. After that surgery our patient started taking oral contraception which she stopped when she decided to have a second pregnancy. The second child was delivered normally. She then resumed taking oral contraception for two years, and stopped. She then started having symptoms of menstrual irregularity and cyclic menstrual pain, but she did not seek any medical help.

**Figure 2:**
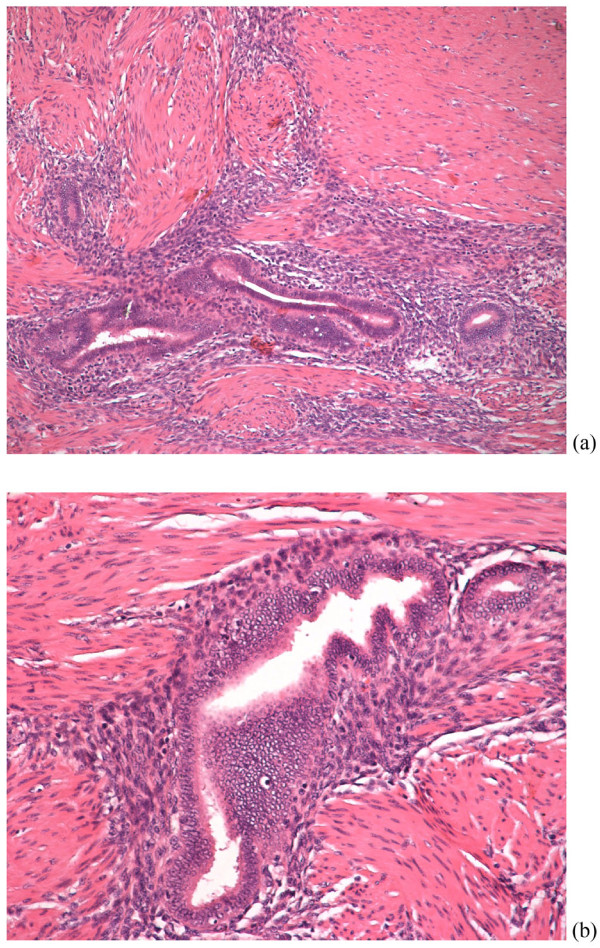
**Endometrial glands and stroma within the muscle coat of the large intenstine (a) H&E×100 (b) H&E×200**.

**Figure 3:**
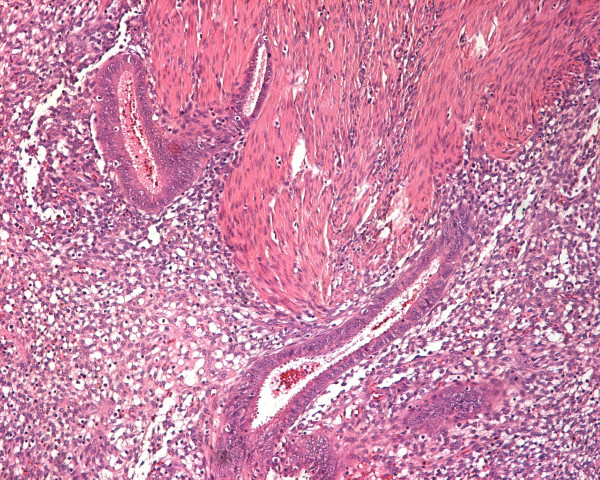
**Endometrial glands and stoma within muscle coat of the appendix (H&E×100)**.

Our patient underwent exploratory laparotomy because of the bowel obstruction. There were adhesions in her abdomen between her appendix, uterus, the sigmoid and her left ureter. Her appendix was found with small abnormal lesions, and an appendectomy was performed. During adhesiolysis, the left ureter was injured, and it was restored at this point using a pig-tail. Her bowel was swollen and the feces could not pass through. The rectum stenosis (almost complete obstruction) was located 8-10 cm from her anus. A rectosigmoidectomy with rectal stump closure and temporary colostomy (Hartmann's procedure) was performed. Our patient recovered and was sent home. The pathology report showed endometriosis of the colon and the appendix (Figures 2, 3). Because of the double location, the gynecologist prescribed medical treatment with triptorelin 3.75 mg every 28 days.

Six months after surgery our patient had another operation for the restoration of large bowel continuity. Her bowel was checked and a specimen was sent to the pathologist. No endometriosis was found. Our patient was doing well at the one-year follow up.

## Discussion

Although endometriosis of the gastrointestinal tract was described by Marshak and Friedman and Grimes in the 1950 s [9,10], its diagnosis is still very difficult, particularly when it appears as acute obstruction of the abdomen.

There are many theories which have tried to explain the pathogenesis of endometriosis, such as Sampson's theory of retrograde spread, vascular dissemination, colonic metaplasia, autoimmune disease, and others [11]. The ectopic endometrial tissue and its behavior can also be explained by these theories and the effect of the ovarian hormones [12]. The progressing processes taking place explain the fibrotic change in the wall of the bowel and the obstruction [12].

The symptoms of intestinal endometriosis vary according to the site of involvement. Τhe symptoms of bowel endometriosis are usually abdominal pain, nausea, vomiting, fecal tenesmus, painful defecation, alternating constipation and diarrhea distention, and rectal bleeding [13,14]. It has to be mentioned that the endometrial foci might have malignant transformation [15].

It is known that the occurrence of endometriosis in the general female population is lower than in that of reproductive age, while the occurrence in endopelvic and gastrointestinal locations is at 5-15% of patients of reproductive age [5,6,16,17]. Gastrointestinal endometriosis (3-37% of an ectopic location) may affect the ileum, appendix, sigmoid colon and rectum [6] with a more frequent location in the rectosigmoid (50-90%). Of the patients with a rectosigmoid location, only a few cases have been reported as an acute abdomen [6,7]. In a large series of patients with endometriosis, the number with intestinal endometriosis and obstruction was between 0.1% and 0.7% [18,19]; therefore, the present case still remains very interesting, because of the diagnostic difficulties.

Intestinal endometriosis often presents as a sub-mucosal tumor or luminal stenosis, because it mainly involves the muscularis propria and subserosa or mesentery. Diagnostic pre-operative evaluation in patients with chronic symptoms is easier and should include computed tomography (CT) scans, magnetic resonance imaging (MRI), and a positron emission tomography (PET) scan to avoid false positive diagnosis of malignancy [20]. Due to the emergency of our case, no CT scan was performed. Another diagnostic problem is the intact mucosa and annular lesion in the bowel wall in the case of diagnostic endoscopy. In the present case, endometriosis was presented as acute abdomen with rectosigmoid obstruction, so our patient was treated as an emergency case with no diagnostic endoscopy. Therefore, in emergency cases like ours, surgical treatment should be considered even when the differential diagnosis of malignancy is not certain.

The histological changes of the intestine wall involving endometriosis are located between muscular fibers, subserosa, and serosa, and the mucosa is mostly intact. In our patient, the pathologist findings showed the annular lesion of endometriosis and the intact mucosa.

Our patient also had endometriosis of the appendix, although the predominant symptoms were only from the rectosigmoid obstruction. Endometriosis of the appendix occurs in approximately 2.8% of patients suffering from endometriosis, and may present with chronic pelvic pain or with symptoms of acute appendicitis [8,21]. In our patient, chronic pain was present but it was not the main symptom.

The treatment of patients with endometriosis is, in general, conservative (for example oral contraceptive, danazol, gonadotropin-releasing hormones, prostaglandin inhibitors) in order to ameliorate the symptoms. Intestinal endometriosis is difficult to diagnose when it presents as an obstruction of the large bowel. It is also difficult to differentiate it from malignancy. In the case of an obstruction, the treatment is surgical with removal of ectopic endometrial tissue, followed by confirmation of free margins.

## Conclusions

Endometriosis of the bowel may present as a large bowel obstruction. To get the diagnosis is difficult because of the limited diagnostic procedures. In our case, final diagnosis could only be given by the pathologist report. In women of reproductive age, the surgeon should consider endometriosis as a differential diagnosis in cases of various gastrointestinal symptoms. Therefore, multidisciplinary care should be encouraged to ensure correct evaluation and improve the management of these patients.

## Consent

Written informed consent was obtained from the patient for publication of this case report and any accompanying images. A copy of the written consent is available for review by the Editor-in-Chief of this journal.

## Competing interests

The authors declare that they have no competing interests.

## Authors' contributions

NK, AKT, KD and CES were the surgical team. ES performed the pathological examination. AKT was the major contributor in writing the manuscript. All authors read and approved the final manuscript.
